# Soft-bottom macrobenthic communities of the coastal marine ecosystem of Chrysi Island (southeast coast of Crete, Greece)

**DOI:** 10.3897/BDJ.14.e202544

**Published:** 2026-07-22

**Authors:** Maria Maidanou, Giorgos Chatzigeorgiou, Panayota Koulouri

**Affiliations:** 1 Hellenic Centre for Marine Research (HCMR), Institute of Marine Biology, Biotechnology & Aquaculture (IMBBC), Heraklion, Crete, Greece Hellenic Centre for Marine Research (HCMR), Institute of Marine Biology, Biotechnology & Aquaculture (IMBBC) Heraklion, Crete Greece

**Keywords:** Chrysi Island, macrobenthos, Marine Protected Area, Mediterranean Sea, soft-bottom sediments

## Abstract

**Background:**

Chrysi Island, an area also including the island of Mikronisi, is located in the southeast of Crete (Greece, eastern Mediterranean). It is a protected ecosystem within the Natura 2000 network (GR4320003), renowned for its diverse marine environment. However, this coastal marine ecosystem, primarily influenced by strong hydrodynamism and characterised by oligotrophy, has never been studied before despite the presence of diverse habitats, such as subtidal rocky reefs associated with communities of macroalgae and sandy soft bottoms covered by *Posidonia oceanica* beds. Amongst the key biological components of this ecosystem, soft-bottom macrobenthic communities are important indicators of environmental stress and human-induced disturbances particularly in the Mediterranean biodiversity and climate-change hotspot.

**New information:**

The present study investigates for the first time the shallow marine environment surrounding the islands of Chrysi and Mikronisi focusing on composition, diversity and distribution patterns of macrobenthic communities inhabiting the sandy soft-bottom sediments, aiming to address one of the important knowledge gaps of the study area. The outcomes of this study are expected to support ongoing conservation efforts and contribute to informed management strategies for safeguarding this coastal marine ecosystem.

A total of eleven sampling stations were established around Chrysi and Mikronisi islands, covering depths ranging from approximately 10 to 30 m and soft substrates consisting mainly of 60 to 90% coarse sand (Koulouri et al. 2023). Sampling was conducted during the spring of 2023 (from 04-23 to 05-01) and funded by the Region of Crete (sub-regional Unit of Lasithi). Soft-bottom sediment samples were collected using standardised benthic sampling procedures and subsequently processed for macrobenthic analysis.

All organisms retained were identified to the lowest possible taxonomic level. In total, 588 individuals were recorded, representing 80 distinct taxa distributed across five major macrobenthic groups: the phyla Annelida, Mollusca, Arthropoda, Echinodermata and a group categorised as “Others”. Amongst these, the class of Malacostraca of the phylum Arthropoda emerged as the most abundant and dominant group across all sampling stations.

## Introduction

The Mediterranean Sea - despite its oligotrophic nature - is recognised as one of the world's most significant marine biodiversity hotspots (Coll et al. 2010). However, it faces multiple anthropogenic pressures that threaten biodiversity, disrupt marine ecosystem functioning and impair the capacity of these ecosystems to deliver essential goods and ecosystem services to human societies (Guidetti et al. 2014). Τhe eastern Mediterranean Sea, in particular, experiencing climate change–driven shift of native marine biodiversity, lacks of baseline biodiversity data, which are essential for assessing ecological conditions and supporting long-term monitoring efforts (Steger et al. 2024).

Chrysi and Mikronisi are small uninhabited islands, located approximately 15 km south of Ierapetra (Prefecture of Lasithi, east of Crete Island, eastern Mediterranean Sea). The islands and surrounding marine environment are designated as a Natura 2000 site (GR4320003). As two of the southernmost landmasses in Greece, they are characterised by warm, oligotrophic and highly transparent waters, typical of the eastern Mediterranean Sea (Azov 1991). The slope of the seabed is steep in the southern part of the two islands (10-m isobath at a distance of approximately 500 m from the coast) in contrast to the northern part which displays a large plateau having the 10-m isobath located, approximately 1-1.5 km from the coast (Koulouri et al. 2023).

The shallow coastal marine environment surrounding the two islands (~ 0–30 m), consists of a mosaic of diverse habitats (Koulouri et al. 2023, Skouradakis et al. 2024a, Skouradakis et al. 2024b), such as subtidal rocky reefs associated with communities of macroalgae (< 20 m, Habitat type 1170) and sandy soft bottoms covered by *Posidonia oceanica* (Linnaeus) Delile 1883 (> 10 m) which is a crucial and protected habitat (Habitat type 1120) in the Mediterranean Sea under the European Union’s Habitats Directive (92/43/EEC) (European Economic Community 1992). These habitats support diverse infaunal and epibenthic macrobenthic communities which remain insufficiently studied across many Mediterranean marine areas, particularly in regions exposed to increasing human pressure and rapid ecological shifts (Coll et al. 2010, Bevilacqua et al. 2020, Tuel and Eltahir 2020).

The shallow sublittoral zone (0-30 m) is characterised by a high percentage of coarse sand ranging from ~ 60% to at least 90% of the sediment. This pattern has been attributed mainly to the intense hydrodynamic conditions north and south-southeast of Chrysi and Mikronisi (Koulouri et al. 2023). Τhe pink-red unicellular species *Miniacina miniacea* (Pallas, 1766) of the phylum Foraminifera characterises the sublittoral zone and contributes to the pink colour of the coastal sediment in the study area (Koulouri et al. 2023).

Given the ecological importance of the study area and the need for baseline information to support management of the protected area, this data paper documents the composition, diversity and distribution of macrobenthic communities inhabiting soft-bottom habitats around the islands of Chrysi and Mikronisi. The resulting dataset provides an ecological baseline for future monitoring, impact assessment and adaptive management of this vulnerable coastal marine ecosystem.

## General description

### Purpose

**Figure 1.**
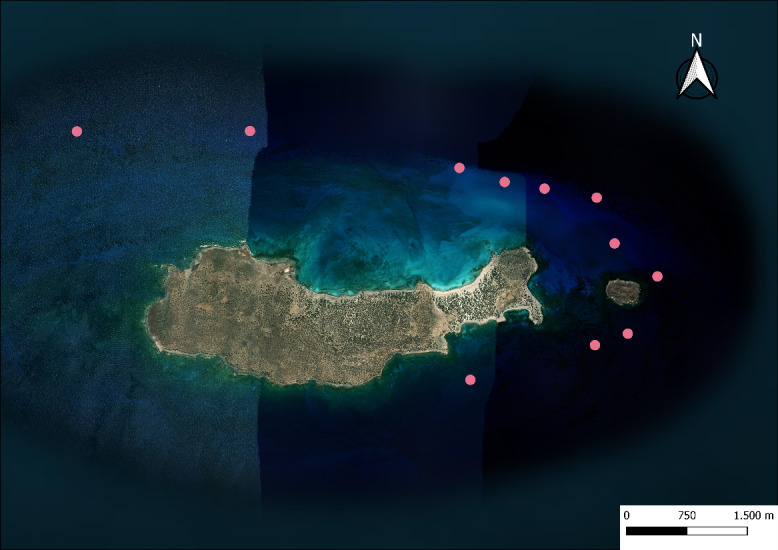
Map of the study area showing sampling stations around Chrysi and Mikronisi islands (Crete, Greece). The satellite image was obtained and modified from Google Earth.

This data paper provides a baseline inventory of the taxonomic composition and abundance of soft-bottom macrobenthic communities in the sublittoral zone, around Chrysi and Mikronisi islands (Fig. 1), based on sediment samples collected between 23 April and 1 May 2023. Eleven stations were sampled at depths of approximately 10 - 30 m with a Smith–McIntyre grab (0.1 m²).

The sediments were dominated by coarse/medium to fine/very fine sand, consistent with the strong hydrodynamic conditions prevailing in the northern and southern parts of the two islands. The dataset forms part of the broader environmental baseline study “Implementation of the Action Plan Measures for Chrysi Island”, funded by the Region of Crete/sub-regional Unit of Lasithi.

## Project description

### Title

Implementation of the Action Plan Measures for Chrysi Island, Region of Crete/sub-regional Unit of Lasithi

### Personnel

Dr Maria Maidanou (sampling, taxonomic identification, data management, manuscript writing), Dr Georgios Chatzigeorgiou (sampling, taxonomic identification, manuscript review), Ioannis Rallis (data curation and management), Dimitra Mavraki (data curation and management), Dr Panayota Koulouri (principal investigator and project manager, expedition design and sampling, taxonomic identification, manuscript writing and editing).

### Study area description

A grid of eleven soft-bottom stations, around Chrysi and Mikronisi islands, (Crete, Greece).

### Design description

This study aimed to enhance understanding of the structure and spatial variability of soft-bottom macrobenthic communities around the marine area of Chrysi and Mikronisi islands.

### Funding

Region of Crete / sub-regional Unit of Lasithi.

## Sampling methods

### Sampling description

A grid of 11 stations was established to assess the qualitative and quantitative composition of the main macrobenthic faunal groups inhabiting soft-bottom substrates. At each station, one sediment sample was collected using a quantitative Smith–McIntyre grab with a sampling surface of 0.1 m². The location of each station was recorded with a FURUNO SFN-70 satellite navigator and water depth was measured using a SIMRAD K-400 echo sounder of the research vessel PHILIA of the Hellenic Centre for Marine Research (HCMR). Samples were sieved on board through a 0.5 mm mesh and preserved in 75% ethanol, buffered with seawater. In the laboratory, macrobenthic organisms were extracted from the sediment, counted and identified to the lowest possible taxonomic level using both a stereoscope and a microscope.

### Quality control

All samples were processed, following a consistent protocol and species identifications were cross-validated by expert taxonomists.

Data management adhered to the FAIR principles (Findable, Accessible, Interoperable, Reusable), ensuring that both metadata and datasets were machine-actionable and appropriately connected through persistent identifiers and established community standards (Wilkinson et al. 2016). Taxonomic and occurrence data were standardised using Darwin Core terms, a globally recognised framework for structuring and exchanging biodiversity information (Wieczorek et al. 2012). Scientific names were harmonised through the World Register of Marine Species (WoRMS) using the Taxon Match tool (WoRMS Editorial Board 2026). Additional measurements and contextual information were standardised using controlled vocabularies from the NERC Vocabulary Server, improving interoperability across marine data repositories (BODC - British Oceanographic Data Centre 2026). Finally, the dataset underwent validation with the LifeWatch & EMODnet Biology Quality Control Tool (Perez et al. 2024), which assesses Darwin Core Archive files against EMODnet Biology data quality criteria.

## Geographic coverage

### Description

The study was conducted across a grid of 11 sampling stations surrounding the islands of Chrysi and Mikronisi, between 10 and 30 m isobaths (Fig. 1). The study site represents the shallow sublittoral zone of the wider area where light penetration supports macroalgal and seagrass habitats and macrobenthic communities are abundant and diverse. The geographical coordinates and depths of the sampling stations are listed in Table 1.

### Coordinates

34.789 and 34.965 Latitude; 25.568 and 25.862 Longitude.

## Taxonomic coverage

### Description

**Figure 2.**
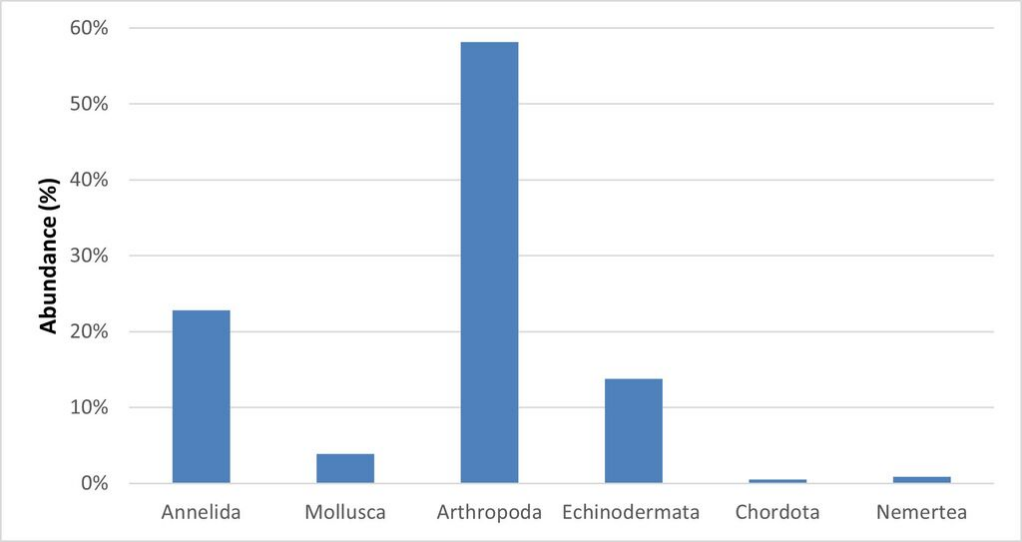
Abundance (%) of the major macrobenthic taxonomic Phyla sampled during the study.

This study provides a baseline assessment of the benthic community structure and diversity within shallow habitats surrounding Chrysi and Mikronisi. A total of 588 individuals were collected and identified, representing 80 distinct macrobenthic taxa. The phylum Arthropoda was the most abundant group, followed by polychaetes and other annelids, echinoderms, molluscs and taxa recorded in relatively low abundances (Chordota and Nemertea), reflecting the complex structure of the benthic assemblage (Fig. 2). The presence of *B. lanceolatum*, a cephalochordate species typically associated with coarse sand and fine gravel substrates (“amphioxus sand”) in the Mediterranean Sea, was recorded in the study area (Dounas 1986, Carlier et al. 2007).

## Temporal coverage

**Data range:** 2023-4-23 – 2023-5-01.

## Collection data

### Collection name

Soft-bottom macrobenthic communities of the shallow coastal marine ecosystem around Chrysi Island (southeast coast of Crete, Greece)

### Specimen preservation method

75% ethanol buffered with seawater.

### Curatorial unit

Hellenic Centre for Marine Research (HCMR), Institute of Marine Biology, Biotechnology and Aquaculture (IMBBC), Heraklion, Crete, Greece.

## Usage licence

### Usage licence

Open Data Commons Attribution License

### IP rights notes

Creative Commons Attribution 4.0 International (CC-BY-4.0).

## Data resources

### Data package title

Soft-bottom macrobenthic communities of the shallow coastal marine ecosystem around Chrysi Island (southeast coast of Crete, Greece)

### Resource link

http://doi.org/10.25607/5iced5

### Alternative identifiers

https://ipt.medobis.eu/resource?r=chrysi-macrobenthos-2023

### Number of data sets

1

### Data set 1.

#### Data set name

Distribution of macrobenthic communities of the coastal marine ecosystem of Chrysi Island (Crete, Greece, spring 2023)

#### Data format

Darwin Core Archive

#### Character set

UTF-8

#### Description

The dataset is available via the MedOBIS (Mediterranean node of Ocean Biodiversity Information System), Integrated Publishing Toolkit (IPT) which has been established through the LifeWatchGreece Research Infrastructure and is hosted in the Institute of Marine Biology, Biotechnology and Aquaculture (IMBBC) of the Hellenic Centre for Marine Research (HCMR). The data are also harvested by and made available through global data repositories such as the Ocean Biodiversity Information System (OBIS). The dataset is available as a DarwinCoreArchive and all fields are mapped to DarwinCore terms (Maidanou et al. 2026).

This publication refers to selected data (Chrysi Island) of the most recent version of the dataset available through MedOBIS.

The current publication refers to the "occurrence" source file (.txt file) that is associated with the particular dataset. While the full dataset reports abundances per 0.1 m² and includes additional replicates, transects and taxa, this data paper focuses specifically on a grid of 11 stations of Chrysi Island. To ensure consistent temporal comparisons of biodiversity, only the macrobenthic phyla for which taxa were identified to species or family level are included (Mollusca, Arthropoda, Annelida, Echinodermata, Others).

Additional details about the sampling events can be found in the "event" source file (.txt file) associated with the same dataset.

**Data set 1.** 

Column label	Column description
id (Event core, Occurrence)	A unique identifier for the record within the dataset or collection, auto-incrementing number automatically added by the system, different between event core and occurrence.
eventType (Event core)	The nature or genre of the resource – here Expedition.
language (Event core)	The language of the resource – here English.
licence (Event core)	A legal document giving official permission to do something with the resource – here CC BY 4.0.
rightsHolder (Event core)	A person or organisation owning or managing rights over the resource – here Hellenic Centre for Marine Research (HCMR).
institutionID (Event core)	An identifier for the institution having custody of the object(s) or information referred to in the record – here https://ror.org/038kffh84.
institutionCode (Event core, Occurrence)	The acronym in use by the institution having custody of the object(s) or information referred to in the record – here HCMR - IMBBC.
eventID (Event core, Occurrence)	An identifier specific to the dataset associated with each event.
eventDate (Event core)	The date during which the event occurred.
habitat (Event core)	A category of the habitat in which the event occured – here sediment type according to EUNIS habitat classification.
habitatID (event core)	The code of the habitat type according to EUNIS habitat classification.
samplingProtocol (Event core)	The descriptions of the methods used during a sampling event – here Smith-McIntyre grab (SMI).
sampleSizeValue (Event core)	A numeric value for a measurement of the sample size – here 0.1 m² (surface area of sampler).
sampleSizeUnit (Event core)	The unit of measurement of the sample size – here representing the surface area of the sampler in m².
locationID (Event core)	An identifier for the set of locality information – here based on the Marine Gazetteer Placedetails.
continent (Event core)	The name of the continent in which the event occurred – here Europe.
country (Event core)	The name of the country or major administrative unit in which the event occurred – here Greece.
countryCode (Event core)	The standard code for the country in which the event occurred – here GR.
locality (Event core)	The specific description of the place – here Chrysi Island.
minimumDepthInMetres (Event core)	The lesser depth of a range of depth below the local surface, in metres.
maximumDepthInMetres (Event core)	The greater depth of a range of depth below the local surface, in metres.
specificEpithet (Occurrence)	The name of the first or species epithet of the scientificName.
decimalLatitude (Event core)	The geographic latitude of the geographic centre of the event (in decimal degrees, WGS84).
decimalLongitude (Event core)	The geographic longitude of the geographic centre of the event (in decimal degrees, WGS84).
geodeticDatum (Event core)	The ellipsoid, geodetic datum or spatial reference system (SRS) upon which the geographic coordinates are based – here WGS84.
georeferenceProtocol (Event core)	A description or reference to the methods used to determine the coordinates – here GPS.
datasetName (Occurrence)	The name identifying the dataset from which the record was derived.
ownerInstitutionCode (Occurrence)	The acronym in use by the institution having ownership of the object(s) referred to in the dataset – here HCMR - IMBBC.
basisOfRecord (Occurrence)	The specific nature of the data record – here PreservedSpecimen.
occurrenceID (Occurrence)	Unique identifier for each occurrence record.
individualCount (Occurrence)	The number of individuals present at each sample.
occurrenceStatus (Occurrence)	A statement about the presence or absence of an occurrence – here only presence.
disposition (Occurrence)	The current state of the sample – here in collection.
verbatimIdentification (Occurrence)	A string representing the taxonomic identification as it appeared in the original record.
scientificNameID (Occurrence)	An identifier for the nomenclatural (not taxonomic) details of a scientific name.
scientificName (Occurrence)	The scientific name.
taxonRank (Occurrence)	The taxonomic rank of the most specific name in scientificName – here species, genus, family.
family (Occurrence)	The full scientific name of the family in which the taxon is classified.
scientificNameAuthorship (Occurrence)	The authorship information scientificName.
genus (Occurrence)	The full scientific name of the genus.
kingdom (Occurrence)	The full scientific name of the kingdom in which the taxon is classified – here Animalia.
phylum (Occurrence)	The full scientific name of the phylum or division in which the taxon is classified.
class (Occurrence)	The full scientific name of the class in which the taxon is classified.
order (Occurrence)	The full scientific name of the order in which the taxon is classified.

## Figures and Tables

**Table 1.** Geographic coordinates (WGS84) and depths (m) of the sampling stations around Chrysi and Mikronisi islands. The geographic coordinates are presented in decimal degrees.

Station	Average Depth (m)	Latitude	Longitude
Chrysi_1	12	34.881497	25.742922
Chrysi_2	19	34.886600	25.740489
Chrysi_3	17	34.887611	25.733400
Chrysi_4	10	34.888353	25.727981
Chrysi_5	13	34.889922	25.721833
Chrysi_6	29	34.894031	25.693367
Chrysi_7	18	34.893994	25.669850
Chrysi_8	18	34.866286	25.723319
Chrysi_10	20	34.877822	25.748736
Chrysi_11	22	34.870156	25.740253
Chrysi_12	20	34.871444	25.744711
